# Left-ventricular diastolic dysfunction in Korean children with chronic kidney disease: data from the KNOW-Ped CKD study

**DOI:** 10.1186/s12882-020-02152-6

**Published:** 2020-12-09

**Authors:** Jeong Yeon Kim, Yeonhee Lee, Hee Gyung Kang, Il-Soo Ha, Hae Il Cheong, Hyun Jin Choi, Kyoung Hee Han, Seong Heon Kim, Min Hyun Cho, Jae Il Shin, Joo Hoon Lee, Young Seo Park, Heeyeon Cho

**Affiliations:** 1 Department of Pediatrics, Samsung Medical Center, Sungkyunkwan University School of Medicine, 81 Irwon-ro, Gangnam-gu, Seoul, 06351 South Korea; 2 Department of Pediatrics, Seoul St. Mary’s Hospital, College of Medicine, The Catholic University of Korea, Seoul, South Korea; 3 Department of Pediatrics, Seoul National University Children’s Hospital, Seoul, South Korea; 4 National Institute of Food and Drug Safety Evaluation, Ministry of Food and Drug Safety, Chungcheongbuk-do, South Korea; 5 Department of Pediatrics, Jeju University Hospital, Jeju, South Korea; 6 Department of Pediatrics, Pusan National University Children’s Hospital, Yangsan, South Korea; 7 Department of Pediatrics, Kyungpook National University School of Medicine, Daegu, South Korea; 8 Department of Pediatrics, Yonsei University College of Medicine, Severance Children’s Hospital, Seoul, South Korea; 9 Department of Pediatrics, Asan Medical Center Children’s Hospital, University of Ulsan College of Medicine, Seoul, South Korea

**Keywords:** Chronic kidney disease, Left ventricular diastolic dysfunction, Children

## Abstract

**Background:**

Cardiovascular disease (CVD) is the most common cause of mortality in pediatric chronic kidney disease (CKD) patients. Left ventricular (LV) hypertrophy (LVH) is associated with LV diastolic dysfunction (LVDD) development and is used as an early marker of CVD in pediatric CKD. This study aimed to assess the prevalence and risk factors of LVDD and the association between LVH and LVDD in Korean pediatric CKD patients.

**Methods:**

Data were collected using the baseline data of the Korean cohort study for outcome in patients with pediatric chronic kidney disease, a nationwide, 10-year, prospective, observational cohort study of pediatric CKD. A total of 244 patients were included in the final analysis. Two-dimensional echocardiography and tissue Doppler images were used to evaluate LVH and LVDD. LVH was defined as an LV mass index (LVMI) ≥38 g/m^2.7^ and LV-wall thickness *z*-score > 1.64. LVDD was defined as a mitral peak velocity of early filling to early diastolic mitral annular velocity (E/E’) > 14. Univariate and multivariate logistic regression analyses were performed to evaluate risk factors of LVDD.

**Results:**

In this study, the male-to-female ratio was 2.2 (168:76) and median age was 11.2 years. The average estimated glomerular filtration rate was 57.4 ml/min/1.73 m^2^, and no patients received renal replacement therapy. The mean value of LVMI and E/E’ was 37.0 g/m^2.7^ and 7.4, respectively. The prevalence of LVH was 40.1 and 17.4% by LVMI ≥38 g/m^2.7^ and LV-wall thickness *z*-score, respectively. The prevalence of LVDD was 4.5%, and patients with LVH showed greater risk of LVDD (odds ratio 7.3, *p* = 0.012). In the univariate analysis, young age, low hemoglobin level, higher LVMI, and higher LV-wall thickness *z*-score were associated with LVDD. In the multivariate analysis, young age, low hemoglobin level, and higher LV-wall thickness *z*-score were independently associated with LVDD.

**Conclusion:**

This study shows that LVH patients have a greater risk of LVDD and that anemia is the only modifiable risk factor for LVDD in Korean pediatric CKD patients.

## Background

Chronic kidney disease (CKD) is an increasing public health issue, and the prevalence of CKD in adults is estimated to be 13.4% worldwide [1]. The prevalence of pediatric CKD is 15–74.7 patients per 1 million children and increased significantly until the first decade of the twenty-first century when treatment and survival improved [2]. Life expectancy is lower in pediatric CKD patients than in the healthy general population, and cardiovascular disease (CVD) is a leading cause of mortality accounting for 25–50% of death in pediatric CKD [3–6]. Previous studies, including the Chronic Kidney Disease in Children (CKiD) study cohort in the USA and the Effect of Strict Blood Pressure Control and ACE Inhibition on the Progression of CRI in PEdiatric Patients (ESCAPE) and the Cardiovascular Phenotypes in Children with CKD (4C) studies in Europe, have shown that early alterations in cardiovascular structure and function occur even before the need for renal replacement therapy [7–9].

In CKD patients, left ventricular (LV) geometry and diastolic function are changed at early stages while systolic functions are preserved until the late stage. These changes are represented as LV hypertrophy (LVH) and diastolic dysfunction (LVDD) by echocardiography and used as an early marker of CVD [9, 10]. However, there is no gold standard method for defining LVH and LVDD in children. In several CKD studies, LV mass index (LVMI) by height^2.7^ (m^2.7^) and the LV-wall thickness *z*-score were used to define LVH with cut-off values of ≥ 38 g/m^2.7^ and > 1.64 for LVMI and *z*-score, respectively [11–13]. LVDD can be measured by the peak of early diastolic flow velocities (E), peak of late diastolic flow velocities (A) by conventional echocardiography, early diastolic peak filling velocity (E’), and late diastolic peak filling velocity (A’) by tissue Doppler echocardiography. The ratio E to E’ is a reliable indicator of diastolic dysfunction and a value over 14 indicates LVDD in adults [14–16]. However, there is no determined cut-off value to define LVDD in pediatric CKD patients. This lack of information makes it difficult to analyze the prevalence and clinical characteristics of pediatric CKD patients with LVDD.

The aim of this study is to detect the prevalence of LVDD in Korean pediatric CKD and the clinical difference according to the presence of LVDD. In addition, as there is no gold standard method for defining LVDD, we are suggesting a cut-off value of E/E’ normalized to the age-independent *z*-score to define LVDD in pediatric CKD patients.

## Methods

### Study design and population

We used the baseline data of the KoreaN cohort study for Outcome in patients With Pediatric Chronic Kidney Disease (KNOW-Ped CKD), a nationwide, 10-year, prospective, observational cohort study of pediatric CKD. The design and methods of KNOW-Ped CKD are published [17]. The study protocol was approved by the Institutional Review Boards of participating clinical centers in 2011. A total of 469 pediatric patients with CKD were registered from the seven major pediatric nephrology centers in Korea. The LVH and LVDD data from echocardiography was available in 395 and 244 patients, respectively. Finally, 244 pediatric CKD patients were included in this study.

### Ethical statement

All the data were obtained in accordance with the ethical principles for medical research involving human subjects established in the Declaration of Helsinki 1975 (revised in 2000).

### Clinical and laboratory measurements

Demographic data and laboratory values were obtained from the web-based data management system, PhactaX (version 1.0). Biochemical values were measured at the hospital laboratories of participating centers. Estimated glomerular filtration rate (eGFR) was calculated using the modified Schwartz equation [18]. The CKD stage was defined according to the Kidney Disease Improving Global Outcome (KDIGO) criteria [19]. The biochemical values were measured at each participating hospital laboratory and additional serum and urine samples were collected for the central laboratory (Lab genomics, Korea). In the central laboratory, serum creatinine (Cr), intact parathyroid hormone (iPHT), 25-OH vitamin D, and 1,25-OH vitamin D levels were measured.

The hypertension was defined when systolic or diastolic blood pressure was above age-, sex-, and height-specific 95th percentile or when the patient was on anti-hypertensive medication. Two-dimensional echocardiography and tissue Doppler were performed by pediatric cardiologists at each center to measure cardiac parameters according to the American Society of Echocardiography pediatric guidelines [16]. The left ventricular mass index was measured from the M-mode measurement of the interventricular septum (IVS), LV inner dimension (LVID), and LV posterior wall thickness (LVPW) using the Devereux formula [20]. LVH was defined in two ways using the LV mass in grams divided by the height in meters to the 2.7th power ≥ 38 g/m^2.7^ (LVH 1) or a height-specific normalized LV wall thickness *z*-score > 1.64 (LVH 2). Early diastolic velocity ratio (E/E’) was evaluated by measuring early mitral valve ventricular filling velocity (E) in conventional echocardiography and early diastolic annular velocity (E’) in tissue Doppler echocardiography. The LVDD was defined using an E/E′ > 14 by tissue Doppler imaging. The E/E’ ratio was normalized to patient age by *z*-score calculation using a published reference based on 325 healthy children [21].

### Statistical analysis

Statistical analysis was conducted by SPSS Version 25. The continuous variables with normal distribution are described as the mean ± standard deviation. The continuous variable with non-normal distribution are expressed as mean ± standard deviation, or median value with interquartile range, whichever describes the data well. A Student’s t-test was used for normally distributed numerical data and Mann–Whitney test was used for non-normally distributed numerical data. Logistic regression analysis was used to evaluate the odds ratio (OR) and confidence interval (CI) for the presence of LVDD associated with each variable. Variable with *P* < 0.1 from univariate analysis were entered in multivariate regression analysis. A chi-squared test and Fisher’s exact test were used for categorical data. The Youden’s index analyzed by empirical method was used to determine the cut-off value for LVDD by E/E’ *z*-score as it showed normal distribution.

## Results

### Participant baseline characteristics and prevalence of LVDD

Demographic and clinical data for 244 patients are listed in Table 1. The median age was 11.2 years old and males was two times more prevalent than females in this cohort. The mean eGFR value was 57.4 and half of the patients were CKD stage III or IV. The patients showed a low z-score of body weight, height, and body mass index (BMI). In 157 patients (71.4%) had hypertension at baseline. In 141 patients (58.3%) who received anti-hypertensive medications, 35 patients had a combination of antihypertensive medications (2 medications, *n* = 28; 3 medications, *n* = 7). Angiotensin-converting-enzyme inhibitors (ACE inhibitors) or angiotensin II receptor blockers (ARBs) were taken in 137 (97.2%) patients and other medications were taken in 4 patients (2.8%). LVMI data showed a mean value of 37.0 g/m^2.7^ (LVMI/height^2.7^) and mean LV wall thickness z-score of -2.9. The prevalence of LVH was 40.1% based on LVMI ≥ 38 g/m^2.7^ and 17.4% based on LV wall thickness z-score > 1.68. Measured for LVDD evaluation, the mean value of E/E’ ratio and E/E’ z-score was 7.4 and 1.3, respectively. The prevalence of LVDD was 4.5% based on E/E’ ratio > 14.

**Table 1:** Demographic and clinical data in cohort patients

Variables	Entire patient (*n* = 244)
Age (years)	11.2 (0.3–17.9)
Sex (Male/Female)	168/76
Body weight, z-score	− 0.8 ± 1.8
Height, z-score	− 0.9 ± 1.6
Body mass index, z-score	−0.3 ± 1.4
Chronic kidney disease stage	
I	45 (18.4)
II	47 (19.3)
III	85 (34.8)
IV	45 (18.4)
V	22 (9.0)
Hypertension (*n* = 220)	157 (71.4)
Systolic blood pressure > 95 percentile (*n* = 204)	27 (13.2)
Diastolic blood pressure > 95 percentile (*n* = 204)	47 (23.0)
Overall blood pressure > 95 percentile (*n* = 204)	58 (28.4)
Patients receiving antihypertensive medications (*n* = 242)	141 (58.3)
Angiotensin-converting-enzyme inhibitors or angiotensin II receptor blockers	137 (97.2)
Other medications	4 (2.8)
Laboratory data	
Hemoglobin (g/dL)	12.4 ± 1.8
Estimated glomerular filtration rate (ml/min/1.73 m^2^)	57.4 ± 38.2
Serum Ca (mg/dL)	9.5 ± 0.7
Serum P (mg/dL)	4.8 ± 0.8
Intact parathyroid hormone (pg/mL)	55.2 (5.8–1244.0)
Echocardiography	
Left ventricular mass index (g/m^2.7^)	37.0 ± 13.0
Patients with left ventricular hypertrophy	97/242 (40.1)
Early diastolic velocity ratio (E/E’)	7.4 ± 3.0

Data are presented as the mean ± standard deviation, as the median with the full range given in the parentheses, or as the number of patients with the percentage given in parentheses

### Calculation of optimal E/E’ ratio value for LVDD

**Fig. 1:**
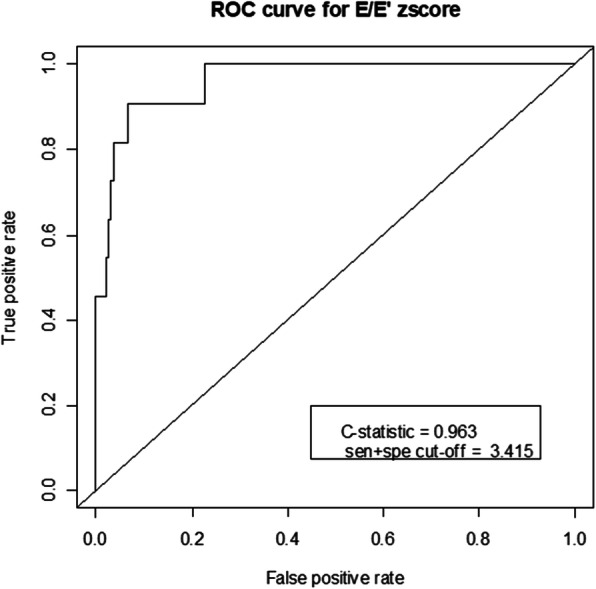
Receiver operating characteristic curve to estimate the optimal E/E’ z-score cut-off value

To estimate the optimal E/E’ ratio reflecting LVDD in children, we normalized the E/E’ ratio to patient age by calculating the z-score based on published reference data from a cohort of 325 healthy children. Based on the definition of LVDD with a measured E/E’ ratio above 14, the E/E’ z-score optimal cut-off value was estimated by Youden’s index to be 3.415 for predicting LVDD (C-statistic = 0.963, sensitivity/specificity = 0.909/0.9313) (Fig. 1). According to newly suggested E/E’ z-score cut-off value, 26 patients (10.7%) were classified as LVDD.

### Clinical difference between patients with and without LVDD

The comparisons between groups with or without LVDD based on an E/E’ ratio > 14 is shown in Table 2. There was no gender difference between groups and more than half of CKD patients with LVDD were early stage CKD (stage 1 and 2). The patients with LVDD were younger (*p* = 0.037) than those without LVDD. The values of LVMI and LV wall thickness *z*-score were higher in CKD patients with LVDD (*p* = 0.002 and *p* = 0.008) compared with those without LVDD. There were no differences in the levels of serum calcium, serum phosphorus, and calcium x phosphorus product and hypertension prevalence between groups.

**Table 2:** Clinical differences in pediatric CKD patients with and without left ventricular diastolic dysfunction

Variable	Patients with left ventricular diastolic dysfunction (*n* = 11)	Patients without left ventricular diastolic dysfunction (*n* = 233)	*P* value
Age, median (full range)	7.6 (0.5–13.0)	11.4 (0.3–17.9)	0.037
Male/Female	6/5	162/71	0.325
Chronic kidney disease stage			
I	4 (36.4)	41 (17.6)	
II	3 (27.3)	44 (18.9)	
III	2 (18.2)	83 (35.6)	
IV	2 (18.2)	43 (18.5)	
V	–	22 (9.4)	
Height Z-score	−1.2 ± 1.0	−0.8 ± 1.6	0.445
Weight Z-score	−1.1 ± 1.1	−0.8 ± 1.8	0.332
Body mass index Z-score	−0.3 ± 1.2	−0.3 ± 1.4	0.861
Estimated glomerular filtration rate (ml/min/1.73 m^2^)	76.7 ± 46.0	56.5 ± 37.7	0.117
Hypertension	6 (66.7)	151 (71.6)	0.718
Hemoglobin (g/dL)	11.1 ± 1.8	12.4 ± 1.8	0.015
Transferrin Saturation (%)	27.6 ± 21.1	27.8 ± 13.0	0.589
Serum albumin (g/dL)	4.1 ± 0.4	4.1 ± 0.6	0.353
Serum Ca (mg/dL)	9.2 ± 0.5	9.5 ± 0.7	0.062
Serum P (mg/dL)	4.8 ± 0.5	4.8 ± 0.9	0.944
Ca x P (mg^2^/dL^2^)	43.9 ± 5.4	45.5 ± 8.5	0.456
Serum tCO2 (mmol/L)	23.3 ± 3.8	22.5 ± 3.5	0.715
Total cholesterol (mg/dL)	154.3 ± 20.7	184.5 ± 70.4	0.054
Serum low-density lipoprotein cholesterol (mg/dL)	95.0 ± 17.2	106.1 ± 63.5	0.888
Serum high-density lipoprotein cholesterol (mg/dL)	46.3 ± 11.9	54.4 ± 15.0	0.077
Serum triglyceride (mg/dL)	108.6 ± 37.5	153.5 ± 158.9	0.347
Intact parathyroid hormone (pg/mL)	49.8 (17.1–243.0)	55.3 (5.8–1244.0)	0.926
25 (OH) vitamin D_3_ (ng/mL)	26.0 ± 17.3	21.1 ± 9.2	0.452
Tubular reabsorption of phosphate (%)	90.5 ± 8.3	77.6 ± 13.7	0.009
Left ventricular mass index (g/m^2.7^)	48.8 ± 14.9	36.5 ± 12.7	0.002
left ventricular wall thickness z-score	1.4 ± 3.7	−3.1 ± 6.3	0.008

Data are presented as the mean ± standard deviation, as the median with the full range given in the parentheses, or as the number of patients with the percentage given in parentheses

### Contributing factors for LVDD

LVDD, defined by measured value of E/E’ > 14, was associated with younger age (*p* = 0.046), low hemoglobin level (*p* = 0.018), increased LVMI/height^2.7^ (*p* = 0.006), and increased LV-wall thickness z-sore (*p* = 0.011) by univariate logistic regression. However, there was no association between LVDD and hypertension. Even though the prevalence of LVH was not exact according to the different methods of definition, patients with LVDD showed greater risk of LVH (LVH1: OR 7.3, *p* = 0.012; and LVH2: OR 4.4, *p* =0.020).

As LVM is indexed by two methods in this study, we analyzed the contributing factors for LVDD with each method. When LVM was indexed by height^2.7^, low hemoglobin level was independently associated with LVDD by multivariable logistic regression (Table 3). When LVM was indexed as LV-wall thickness z-score, low hemoglobin level, young age, and LV wall thickness z-score were independently associated with LVDD by multivariable logistic regression (Table 4).

**Table 3:** Associated factors of left ventricular diastolic dysfunction with the definition of LVH1

	Unadjusted OR (95% CI)	*P* value	Adjusted OR (95% CI)	*P* value
Age	0.882 (0.78–0.998)	0.046	–	–
Hemoglobin	0.67 (0.481–0.933)	0.018	0.566 (0.373–0.859)	0.007

*LVH1* Left ventricular hypertrophy defined by left ventricular mass in grams divided by height in meter to the 2.7th power ≥ 38 g/m^2.7^; *OR* Odds ratio, *CI* Confidence interval

**Table 4:** Associated factors of left ventricular diastolic dysfunction with the definition of LVH2

	Unadjusted OR (95% CI)	*P* value	Adjusted OR (95% CI)	*P* value
Age	0.882 (0.78–0.998)	0.046	0.659 (0.509–0.853)	0.002
Hemoglobin	0.67 (0.481–0.933)	0.018	0.538 (0.318–0.912)	0.021
LV wall thickness z-score	1.197 (1.043–1.373)	0.011	1.537 (1.225–1.928)	0.000

*LV* Left ventricular, *LVH2* height-specific normalized LV wall thickness *z*-score > 1.64, *OR* Odds ratio, *CI* Confidence interval

LVDD, defined by newly suggested E/E’ z-score > 3.415, was associated with increased LV-wall thickness z-sore (OR 1.2, *p* = 0.002), presence of LVH2 (OR 4.7, *p* = 0.0005), and not with age by univariate logistic analysis. Only presence of LVH2 showed association with LVDD in multivariable logistic regression (OR 4.0, *p* = 0.0038) (Table 5).

**Table 5:** Associated factors of left ventricular diastolic dysfunction define by E/E’ ratio z-score > 3.415 with the definition of LVH2

	Unadjusted OR (95% CI)	*P* value	Adjusted OR (95% CI)	*P* value
LV wall thickness z-score	1.173 (1.060–1.298)	0.0020	–	–
LVH2	4.714 (1.962–11.326)	0.0005	4.04 (1.57–10.4)	0.0038

*LV* Left ventricular, *LVH2* height-specific normalized LV wall thickness *z*-score > 1.64, *OR* Odds ratio, *CI* Confidence interval

## Discussion

The LVH is a well-known LV geometry change in pediatric CKD patients, which progress as CKD stage advance and showed association with hyperparathyroidism, vitamin D deficiency, anemia, and hypertension in previous studies [22, 23]. Previously, unlike systolic dysfunction presented in adult progressed CKD patients, in pediatric CKD patients, the systolic function was presumed preserved [24]. However, further research revealed that subclinical systolic dysfunction (abnormal systolic walls mechanics with preserved LV chamber function) exists in pediatric CKD patients [25]. Even though the hypertension was thought to be the main factor which affects the LVH progression in pediatric CKD patient, it was often under and even untreated [26]. In the study conducted by Matteucci et al. revealed that strictly controlled blood pressure showed regression of LVH and improvement of subclinical systolic function in pediatric CKD patients expected to result in cardiovascular outcome improvement [27]. Also, in other studies, not only hypertension but also cardiac output increment and nonhemodynamic factors showed an association with the prevalence of LVH in pediatric CKD patients [28, 29]. Therefore, like LVH, it is also crucial to find modifiable factors associated with LVDD in CKD patients to improve their cardiovascular outcomes by improving LVDD. However, there is a lack of data regarding LVDD in pediatric CKD patients. The prevalence of LVDD in our study was 4.5%, which was lower compared with previous studies [30]. The low prevalence may be explained in some ways by the characteristics of the enrolled patients and the different definitions of LVDD. The previously reported prevalence of LVDD in pediatric dialysis CKD patients, based on an E/E’ ratio above the maximum value (max E/E’ ratio in healthy control was 11.9) of healthy age- and gender-matched controls, was 29%. As dialysis CKD patients had worse LVDD than pre-dialysis CKD patients, the prevalence of LVDD in overall CKD may be much lower [30]. The increment of LVH prevalence according to progressive CKD stage in children is also well known [31]. Our patients were in pre-dialysis condition, and the evaluation of the secular trend of LVDD prevalence is necessary.

The cut-off value of the E/E’ ratio to define LVDD may have influenced the prevalence in our study. There were several studies in which CKD children have worse LVDD than healthy children, and LVDD worsened as CKD progressed [30, 32]. However, the absence of a consensus standard method for defining LVDD affected the lack of interpretation of LVDD clinical significance in pediatric CKD patients. Children are in the process of growth and which impacts echocardiography parameters as the heart grows [21]. Therefore, the definition of LVH in pediatric patients are indexed by multiple ways including body size, age-specific reference, and the LVM percentile according to height [11, 12, 33]. Like LVH, the LVDD definition needs to be normalized or indexed in pediatric patients. To establish a reference value of Doppler tissue imaging in children, Eiden et al. gathered echocardiography parameters in 325 healthy children (age range: 1 day to 18 years) in the USA. This study found a significant association between E/E’ and age in healthy children [21]. Recently reported data from the 4C study showed impaired systolic and diastolic function in pediatric CKD patients by using normalized echocardiographic parameters to age-independent z-score according to the reference data mentioned above [34]. Likewise, we normalized the E/E’ ratio to age by using the reference value reported by Eiden et al. As we used an E/E’ ratio > 14 based on adult data to define LVDD, we attempted to find an optimal E/E’ z-score cut-off value to define LVDD in pediatric CKD patients by Youden’s index [15, 21, 35]. The estimated cut-off value of the E/E’ z-score was 3.415 in this study. Further studies are needed to validate the impact of LVDD defined by an E/E’ z-score > 3.415 on long term outcomes in pediatric CKD including CVD events. Additionally, since pediatric CKD patients are accompanied by inadequate growth problem, age-independent E/E’ z-score might not be the best way to normalize LVDD in pediatric CKD patients. Like LVH defining methodologies, body size, age-specific indexed E/E’ ratio should be considered in the future.

This study describes the clinical differences associated with the presence of LVDD in pediatric CKD patients. In previous studies, the E/E’ ratio in pediatric CKD showed an association with young age, serum phosphorus level, calcium x phosphorous product, LVMI, and anemia but not with hypertension [10, 30, 32]. Unlike the adult population showing strong relation between LVDD and blood pressure, E/E’ ratio in children showed association only to BMI, waist circumference, obesity, and not to hypertension, based on a large sample study of non-CKD children [36, 37]. The LVDD in this study was associated with young age, low hemoglobin level, and LV wall thickness z-score, not with hypertension, which was similar to previous reports. Additionally, as LVDD develops more in dialysis than non-dialysis CKD patients, volume overload might be a more important factor in developing LVDD in pediatric CKD patients than pressure overload [10]. As LVDD defined by age-standardized E/E’ ratio showed no association with age, previously known contributing factor of younger age to LVDD might be a result of paucity of E/E’ reference value in pediatric population. Still, further validation is required in optimal E/E’ Z-score cut-off value. Among the revealed LVDD contributing factors, only anemia is an adjustable factor in pediatric CKD patients. Further, LVH needs to be close monitored in LV wall thickness z-score. However, LVH defined as an LVMI ≥ 38 g/m^2.7^ and LV wall thickness z-score > 1.64 shows poor concordance and this discrepancy needs to be studied in future research [13].

This study has several limitations. First, echocardiography was performed by pediatric cardiologists in each center without central reading validation, which can show poor concordance between examiners. Second, this study only included data from baseline pre-dialysis CKD patients. Although LVDD may exist in early CKD, it is well known that LVDD worsens as CKD progresses, and a longitudinal study is necessary to evaluate the overall prevalence. Third, the normalized E/E’ z-score was based on a reference from 325 healthy children in the USA. This study includes Korean children, and ethnic differences may influence the results.

## Conclusion

This study showed a 4.5–10.7% prevalence of LVDD in pre-dialysis CKD, which is associated with young age, anemia, and LV-wall thickness z-score. Further, we suggest an E/E’ z-score of 3.415 as optimal cut-off value to define LVDD in pediatric CKD patients.

## Data Availability

The dataset can be available that is within the perspective of the scientific objectives of KNOW-PedCKD and researchers who approved by the KNOW-PedCKD investigators can access the data (http://www.know-pedckd.org/pedckd/main/main.html).

## Abbreviations

CKD: Chronic kidney disease
CVD: Cardiovascular disease
CKiD: Chronic Kidney Disease in Children
ESCAPE: Effect of Strict Blood Pressure Control and ACE Inhibition on the Progression of CRI in PEdiatric Patients
LV: Left ventricular
LVH: Left ventricular hypertrophy
LVDD: Left ventricular diastolic dysfunction
LVMI: Left ventricular mass index
E: Peak of early diastolic flow velocities
A: Peak of late diastolic flow velocities
E’: Early diastolic peak filling velocity
A’: Late diastolic peak filling velocity
KNOW-Ped CKD: KoreaN cohort study for Outcome in patients With Pediatric Chronic Kidney Disease
eGFR: Estimated glomerular filtration rate
KDIGO: Kidney Disease Improving Global Outcome
Cr: Serum creatinine
iPTH: Intact parathyroid hormone
IVS: Interventricular septum
LVID: Left ventricular inner dimension
LVPW: Left ventricular posterior wall thickness
OR: Odds ratio
CI: Confidence interval
